# Effect of Factor XIII-A G185T Polymorphism on Visual Prognosis after Photodynamic Therapy for Neovascular Macular Degeneration

**DOI:** 10.3390/ijms160819796

**Published:** 2015-08-20

**Authors:** Francesco Parmeggiani, Ciro Costagliola, Francesco Semeraro, Mario R Romano, Michele Rinaldi, Carla Enrica Gallenga, Maria Luisa Serino, Carlo Incorvaia, Sergio D’Angelo, Katia De Nadai, Roberto Dell’Omo, Andrea Russo, Donato Gemmati, Paolo Perri

**Affiliations:** 1 Eye Clinic, Department of Biomedical and Specialty Surgical Sciences, University of Ferrara, Via Aldo Moro 8, 44124 Cona-Ferrara, Italy; E-Mails: gllcln@unife.it (C.E.G.); ncrcrl@unife.it (C.I.); das@unife.it (S.D.A.); katia.denadai@libero.it (K.D.N.); prrpla@unife.it (P.P.); 2 Eye Clinic, Department of Health Sciences, University of Molise, Via Francesco de Sanctis 1, 86100 Campobasso, Italy; E-Mails: ciro.costagliola@unimol.it (C.C.); robdellomo@libero.it (R.D.O.); 3 Eye Clinic, Department of Neurological and Vision Sciences, University of Brescia, Piazzale Spedali Civili 1, 25123 Brescia, Italy; E-Mails: francesco.semeraro@unibs.it (F.S.); dott.andrea.russo@gmail.com (A.R.); 4 Eye Clinic, Department of Neuroscience, Reproductive and Odonto-Stomatological Sciences, “Federico II” University of Naples, Via Pansini 5, 80131 Napoli, Italy; E-Mail: mario.romano4@unina.it; 5 Department of Ophthalmology, Second University of Naples, Via Pansini 5, 80131 Napoli, Italy; E-Mail: michele.rinaldi@unina2.it; 6 Center of Hemostasis and Thrombosis, Department of Medical Sciences, University of Ferrara, Corso Giovecca 203, 44121 Ferrara, Italy; E-Mails: srnmls@unife.it (M.L.S.); cet@unife.it (D.G.)

**Keywords:** macular degenerations, choroidal neovascularization, pharmacogenetics, photodynamic therapy with verteporfin, fibrin-clot stability, factor XIII-A G185T gene polymorphism, anti-thrombophilia

## Abstract

Macular degenerations represent leading causes of central blindness or low vision in developed countries. Most of these severe visual disabilities are due to age-related macular degeneration (AMD) and pathologic myopia (PM), both of which are frequently complicated by subfoveal choroidal neovascularization (CNV). Photodynamic therapy with verteporfin (PDT-V) is still employed for CNV treatment in selected cases or in combined regimen. In Caucasian patients, the common polymorphism G185T of factor XIII-A gene (FXIII-A-G185T; rs5985) has been described as predictor of poor angiographic CNV responsiveness to PDT-V. Nevertheless, the prognostic implications of this pharmacogenetic determinant on long-term visual outcome after a PDT-V regimen have not been evaluated. We retrospectively selected Caucasian patients presenting with treatment-naive CNV and receiving standardized PDT-V protocol for two years. The study population included patients affected by subfoveal CNV secondary to AMD or PM. We assessed the correlations between the polymorphic allele T of FXIII-A-G185T and: (1) total number of photodynamic treatments; and (2) change in visual acuity from baseline to the end of the follow-up period. Considering a total study population of 412 patients with neovascular AMD or PM, the carriers of 185 T-allele of FXIII-A (GT or TT genotype) received a higher number of photodynamic treatments than patients without it (GG wild-type genotype) (*p* < 0.01; mean number of PDT-V: 5.51 *vs.* 3.76, respectively). Moreover, patients with 185 T-allele of FXIII-A had a more marked worsening of visual acuity at 24 months than those with the GG-185 wild genotype (*p* < 0.01; mean difference in logMAR visual acuity: 0.22 *vs.* 0.08, respectively). The present findings show that the G185T polymorphism of the FXIII-A gene is associated with significant differences in the long-term therapeutic outcomes of patients treated with standardized PDT-V protocol. The comprehensive appraisal of both anti-thrombophilic effects due to FXIII-A G185T variant and photo-thrombotic action of PDT-V toward CNV provides several clues about the rationale of this intriguing pharmacogenetic correlation. Further investigations are warranted to outline the appropriate paradigm for guiding PDT-V utilization in the course of the combined therapeutic protocol for neovascular macular degeneration.

## 1. Introduction

Macular degenerations represent the leading causes of central blindness or low vision in developed countries. The largest segment of these severe visual impairments is secondary to age-related macular degeneration (AMD) and, to a minor extent, to pathologic myopia (PM), both of which are frequently complicated by choroidal neovascularization (CNV) [1,2,3,4]. The therapeutic management of patients affected by neovascular macular degenerations represents one of the main pharmacoeconomic problems for the Health Systems of Western Europe, North America and Australia [5,6,7]. CNV consists of an invasive vascular complex, able to induce a breakdown of the chorioretinal barrier starting from a degenerated area of retinal pigment epithelium (RPE) [8,9]. The natural history of CNV is usually characterized by the development of aberrant blood vessels under and/or inside the macula, which represents the central portion of the retina and is responsible for high-resolution vision. Two main therapeutic options, both approved by the Food and Drug Administration and by the European Agency for the Evaluation of Medicinal Products, are currently available to counteract the vision loss due to subfoveal CNV: photodynamic therapy with verteporfin (PDT-V); and drugs acting against vascular endothelial growth factor (anti-VEGF) [4,10,11,12,13]. In particular, over the last few years, the anti-CNV therapies evolved from protocols aimed at stabilizing a patient’s vision (*i.e*., quarterly execution of PDT-V procedure) [11,14,15,16,17], to treatments capable of improving visual acuity (*i.e*., monthly or as-needed intravitreal administration of anti-VEGF drugs) [12,13,18,19,20,21,22]. After all, considering not only the visual benefit and its maintenance but also a lower re-treatment frequency and optimization of health resource employment, positive outcomes are observed when PDT-V is combined with anti-VEGF regimens [6,23,24,25,26,27,28,29,30,31,32,33,34].

In neovascular macular degenerations, the therapeutic action of PDT-V is achieved by a laser-light-induced thrombosis of neovascular complex, which has been previously photosensitized by the administration of verteporfin [35,36,37,38,39]. PDT-V for either AMD- or PM-related CNVs is generally performed according to the standardized protocol of the Treatment of Age-Related Macular Degeneration with Photodynamic Therapy (TAP) study and of the Visudyne in Photodynamic Therapy (VIP) study [14,15,16,17]. Several predictors, such as patient’s age, baseline visual acuity, and CNV size at presentation, have been considered to explain the high variability of PDT-V effectiveness in patients with neovascular AMD [40,41,42] or neovascular PM [43,44]. Reviewing the visual outcomes of patients with AMD-related CNV enrolled in TAP and VIP studies, the individually different efficacy of PDT-V is clearly evident. In fact, at the 24-month check, the percentage of eyes with or without severe visual worsening (over three lines of visual acuity loss) appears rather balanced: 41% *vs.* 59% in predominantly classic CNV (TAP study), 55% *vs.* 45% in occult with no classic CNV (VIP study), and 52% *vs.* 48% in minimally classic CNV (TAP study) [10]. Similarly, in the VIP study, the proportion of myopic patients with or without reduction of visual acuity after 24 months of the verteporfin regimen is 46% *vs.* 54% [16]. Moreover, both Chan *et al.* and Yang *et al.* have interestingly pointed out the presence of remarkable differences in CNV responsiveness to standardized PDT-V between Asian and Caucasian patients considering, respectively, all forms of CNV [45] and just those secondary to AMD [46]. Likewise, comparing the rate of vision improvement or stabilization recorded in Caucasians (62%) [47] and in Asians with myopic CNV, the efficacy of PDT-V seems to be markedly greater within both Chinese [48] and Japanese [49] populations (respectively, 80.6% and 85.7%). Short-term investigations in Caucasian patients have indicated that some single nucleotide polymorphisms (SNPs) modulating hemostasis and/or fibrinolysis [50,51,52] may be involved in the above-described disparities [10,14,15,16,45,46,47,48,49]. In fact, taking into account the photo-thrombotic mechanisms of PDT-V [35,36,37,38,39], it is consistent that coagulation-balance SNPs can be predictive of variable angiographic CNV responsiveness to photodynamic treatment [53,54,55,56]. Particularly, the common polymorphism G185T of factor XIII-A gene (FXIII-A-G185T; rs5985) acts as a negative factor for the post-PDT-V occlusion of CNV in both AMD and PM patients [50,51,52]. However, the role of this pharmacogenetic determinant in long-term visual prognosis after PDT-V protocol has not yet been defined. The current study aimed to verify whether, after a two-year period of PDT-V protocol, the carriers of FXIII-A G185T polymorphism with AMD- or PM-related CNV required more photodynamic re-treatments than patients without this genetic variant, and to determine whether modifications in visual acuity differed between these two groups. The confirmation of these work-hypotheses might represent a pharmacogenetic novelty useful to optimize the clinical employment of PDT-V.

## 2. Results

The study population consisted of 412 patients undergoing standardized PDT-V protocol [14,15,16] after the diagnosis of treatment-naive neovascular macular degenerations: 139 cases of classic or predominantly classic AMD-related subfoveal CNV, 105 cases of minimally classic or occult with no classic AMD-related subfoveal CNV, and 168 cases of classic PM-related subfoveal CNV. A total of 59 patients were not considered in the final statistical analyses because they had started antiplatelet, antithrombotic and/or anticoagulant systemic therapies after the first PDT-V procedure. Moreover, other 109 patients were ruled out due to lack of follow-up (49 cases), imprecise adhesion to the check timing (34 cases), or incomplete data collection (26 cases). At baseline, the demographic and clinical attributes of these excluded patients were not statistically different with respect to those of the study cluster.

In the final study population, patients with wild-GG genotype of FXIII-A-G185T polymorphism were 225, whereas 187 were carriers of polymorphic genotypes (GT in 154 cases and TT in 33 cases). The baseline comparisons between demographic and clinical characteristics of FXIII-A 185-T-allele carriers and non-carriers are shown in Table 1; all these attributes were not statistically different within the two allelic groups. No significant deviations from the Hardy-Weinberg equilibrium and genotype distribution were observed comparing FXIII-A-G185T polymorphism among a control group of healthy Caucasian individuals (*n* = 200; GG = 114; GT = 73; TT = 13) [57] and the study group (*n* = 412; GG = 225; GT = 154; TT = 33). The mean number of PDT-V administered through the follow-up period (including the first procedure and the as-needed treatments performed according to TAP/VIP protocol) [17,47] was: 5.08 in patients with classic or predominantly classic AMD-related CNV, 4.62 in patients with minimally classic or occult AMD-related CNV, and 4.17 in patients with classic PM-related CNV. These total amounts of PDT-V resemble those observed in the randomized controlled trials after two years of follow-up [15,16,58,59].

**Table 1:** At-baseline comparisons between demographic and clinical characteristics of factor XIII-A 185-T-allele carriers (patients with GT or TT polymorphic genotypes) and non-carriers (patients with wild-GG genotype).

Study Population (*n* = 412 Patients Affected by Subfoveal CNV)					
Baseline Characteristics	FXIII-A 185-T-Allele Carriers (*n* = 187)	FXIII-A 185-T-Allele Non-Carriers (*n* = 225)	*p* Value
Sex			
Male/Female–no. (%)	86 (46.0)/101 (54.0)	104 (46.2)/121 (53.8)	NS *
Mean age ± SD (range)–years	64.514 ± 13.807 (34–86)	64.802 ± 15.122 (33–88)	NS ^†^
Mean BCVA ± SD (range)–logMAR	0.604 ± 0.215 (0.2–1.0)	0.594 ± 0.231 (0.1–1.0)	NS ^†^
Mean CNV area ± SD (range)–micron^2^	2894.2 ± 2789.3 (232–10,065)	2759.4 ± 2605.7 (244–10,125)	NS ^†^
Type of neovascular lesion			
Classic or predominantly classic AMD-related CNV–no. (%)	63 (33.7)	76 (33.8)	NS *
Minimally classic or occult AMD-related CNV–no. (%)	48 (25.7)	57 (25.3)	NS *
Classic PM-related CNV–no. (%)	76 (40.6)	92 (40.9)	NS *

Legend: CNV, choroidal neovascularization; FXIII, factor XIII; SD, standard deviation; BCVA, best-correct visual acuity; logMAR, logarithm of the minimum angle of resolution; AMD, age-related macular degeneration; PM, pathologic myopia; *, χ*^2^* test; ^†^, two-sided *t*-test; NS, not significant.

At the end of the follow-up period among carriers of 185 T-allele of FXIII-A (GT or TT genotype) the mean number of PDT-V ± standard deviation (SD) was 5.51 ± 1.52 (range: from 1 to 8), whereas in patients with GG wild-type genotype the average of PDT-V ± SD was 3.76 ± 1.49 (range: from 1 to 8). The statistical comparison of these data, controlled for patient’s age, sex and baseline CNV area, demonstrates that carrier of FXIII-A 185-T-allele received a higher number of photodynamic treatments in contrast to non-carriers (*p* < 0.01). In fact, after our long-term monitoring, four or less photodynamic treatments were necessary just in 45 of 187 patients (24.0%) with the polymorphic T-allele, but this low total number of PDT-Vs was sufficient to block CNV activity in 142 of 225 patients (63.1%) without it (Table 2). During the final eye check, the carriers of 185 T-allele of FXIII-A had a worse mean BCVA ± SD in comparison with non-carriers, respectively, 0.81 ± 0.31 logMAR (range: from 0.2 to 1.3) *vs.* 0.59 ± 0.33 logMAR (range: from 0.1 to 1.3) (*p* < 0.01) (Table 2). In addition, patients with GT or TT genotype of FXIII-A G185T polymorphism had a mean reduction in BCVA ± SD of 0.22 ± 0.03 logMAR (range: from −0.3 to 0.7) at month 24, whereas among the GG wild-type patients the BCVA diminution was just equal to 0.08 ± 0.02 logMAR (range: from −0.4 to 0.6). The comparison of these data, controlled for patient’s age, sex and baseline CNV area, was statistically significant (*p* < 0.01) documenting a pharmacogenetic relationship between FXIII-A 185-T-allele and poor visual prognosis after a prolonged anti-CNV photodynamic regimen. Although a post-PDT-V stabilization or improvement of BCVA was recorded just in 44 of 187 patients (23.5%) with FXIII-A G185T polymorphism, this satisfactory result was present in 127 of 225 (56.4%) non-carriers of this gene variant (Table 2).

**Table 2:** Comparative analyses of clinical outcomes between factor XIII-A 185-T-allele carriers (patients with GT or TT polymorphic genotypes) and non-carriers (patients with wild-GG genotype).

Study Population (*n* = 412 Patients Affected by Subfoveal CNV)			
Outcome Measures	FXIII-A 185-T-Allele Carriers (*n* = 187)	FXIII-A 185-T-Allele Non-Carriers (*n* = 225)	*p* Value
Number of PDT-V	No. (%)	No. (%)	
1–2	7 (3.7)	31 (13.8)	
3–4	38 (20.3)	111 (49.3)	
5–6	100 (53.5)	68 (30.2)	
7–8	42 (22.5)	15 (6.7)	
Mean number of PDT-V ± SE	5.51 ± 0.13	3.76 ± 0.12	0.01 *
Final BCVA (logMAR)	No. (%)	No. (%)	
0.1–0.3	8 (4.3)	39 (17.3)	
0.4–0.6	45 (24.1)	78 (34.7)	
0.7–0.9	70 (37.4)	70 (31.1)	
1.0–1.3	64 (34.2)	38 (16.9)	
Mean final BCVA ± SD	0.81 ± 0.31	0.59 ± 0.33	0.01 ^†^
BCVA change (logMAR)	No. (%)	No. (%)	
−0.4–−0.2	12 (6,4)	20 (8.9)	
−0.1–0.0	32 (17.1)	107 (47.5)	
0.1–0.2	22 (11.8)	45 (20.0)	
0.3–0.7	121 (64.7)	53 (23.6)	
Mean BCVA change ± SE	0.22 ± 0.03	0.08 ± 0.02	0.01 *

Legend: CNV, choroidal neovascularization; FXIII, factor XIII; PDT-V, photodynamic therapy with verteporfin; SE, standard error; BCVA, best-correct visual acuity; logMAR, logarithm of the minimum angle of resolution; SD, standard deviation; *, ANOVA test; ^†^, two-sided *t*-test; NS, not significant.

## 3. Discussion

The present study has shown that the G185T polymorphism of FXIII-A gene, already recognized as able to downgrade the short-term angiographic response of subfoveal CNV to PDT-V [50,51,52], is associated with significant differences in the long-term therapeutic outcomes of patients treated with standardized PDT-V protocol for AMD- or PM-related neovascular macular degenerations. In fact, after two years of follow-up, although the carriers of the polymorphic T-allele received more photodynamic treatments than non-carriers, they paradoxically experienced a more remarkable reduction of visual acuity in comparison to patients with the GG-185 wild genotype. Several heterogeneous aspects may be involved in the different CNV responsiveness to PDT-V in each treated patient, especially considering those inherited, environmental and iatrogenic factors able to influence the oxidative and para-inflammatory interactions between each procedure of photodynamic therapy, its target lesion (*i.e*., the subfoveal CNV) and the degenerated retina-RPE tissues in which this neovascular complication occurred [60,61,62,63]. However, an all-embracing appraisal of both the anti-thrombophilic diathesis of the common FXIII-A G185T variant and the distinctive photo-thrombotic action of PDT-V at the level of CNV micro-vasculature, provides a plausible explanation about the rationale of this intriguing pharmacogenetic correlation [35,36,37,38,39,55,56], regardless of other phenotypic and genotypic characteristics [40,41,42,43,44,50,51,52,56].

In neovascular macular degenerations, experimental and clinical findings indicate that the therapeutic effects of PDT-V are mainly determined by three combined mechanisms of action: cellular, vascular, and immunological [35,36,37,38,39]. PDT-V benefits result from a selective shutdown of CNV micro-vasculature with consequent stoppage of the invasive force of the neovascular complex, which has been photosensitized by the intravenous administration of verteporfin just before the laser application. The photo-thrombotic action of PDT-V is due to a preferential binding of this specific photosensitizer to the endothelium of CNV in comparison with that of the normal retino-choroidal vasculature of the macula. In fact, verteporfin couples with low density lipoproteins (LDL) to form a complex which is prevalently up-taken into neovascular endothelial cells owing to their overexpression of LDL receptors. After PDT-V, the changes of neovascular endothelium are caused by the photo-oxidative action of several reactive oxygen species (ROS), which trigger those specific events causative of the therapeutic hemostasis inside CNV. ROS-related exposure of vascular basement membrane activates adhesion, degranulation and aggregation of the platelets, followed by the release of vasoactive mediators. These molecules elicit a cascade of events, *i.e.*, amplification of platelet activation, thrombosis, vasoconstriction, and increased vascular permeability, which lastly cause blood stasis, tissue hypoxia, and CNV occlusion [35]. Although this mechanism of action has been initially considered ideal to obtain the photo-thrombotic blocking of subfoveal CNV, the individually variable efficacy of standardized PDT-V is clearly noticeable reviewing the outcomes of randomized controlled trials [15,16,58,59], as well as those of the real-life clinical practice [64,65].

Coagulation factor XIII plays a pivotal role in formation, arrangement, and stability of the blood clot. In particular, the A subunit of factor XIII catalyzes the cross-linking between the fibrin molecules to make the thrombus more compact and resistant [66,67]. Because of the direct interaction of factor XIII with both fibrinogen and platelets, several abnormalities of fibrin network can be most likely related to changes of factor XIII [68,69]. Concentration, bio-availability and activity level of factor XIII strongly depend from specific intra-genic polymorphisms [70]. In particular, the G-to-T transversion at nucleotide 185 of FXIII gene underlying the replacement of a valine by a leucine at codon 34 (Val34Leu) in the catalytic A subunit of factor XIII, is considered the main functional locus of this coagulation factor [71]. In fact, the mutant 34Leu form of this activation-peptide generates an anti-thrombophilic status by means of direct or indirect effects on: (1) factor XIII transglutaminase activity [72,73]; (2) thrombin-related activation of factor XIII [73,74]; (3) tridimensional structure of the cross-linked fibrin-clot [71,75,76,77,78,79].

Polymorphic GT and TT genotypes are associated with important modifications in enzymatic function of factor XIII, which is markedly increased in homozygotes and exhibits an intermediate activity in heterozygous carriers [68,69,72,76]. Especially when plasmatic level of fibrinogen becomes abnormally elevated, as locally occurs during PDT-V [35], the fibrin fibers cross-linked by the 34Leu variants are thicker and more loosely packed than those formed by the wild 34Val variant [55,77,78].

In patients affected by subfoveal CNV, the retinochoroidal angiograms performed after PDT-V reveal a massive fluid extravasation at the level of the irradiated macular area, that forbid an exact assessment of the extent of CNV occlusion before the slow regression of these exudative phenomena in the course of the next 6 to 7 days [39]. On the other hand, the timing of the hemodynamic closure of CNV is variable and, at the 7-day check after PDT-V, the angiography shows signs of active neovascular net in about 50% of the treated patients. [38] This aspect suggests that the early CNV recanalization can represent a crucial cause of poor responsiveness to photodynamic protocol. The worse clinical outcomes, observed at the end of PDT-V protocol among our carriers of FXIII-A 185-T-allele, can be rationally related to a weak photo-thrombotic effect inside the neovascular target of these therapeutic procedures [35,55,56,76,77,78]. In the post-PDT-V condition of elevated fibrinogenemia, the enlarged space between the fibrin strands inside the clot of patients with FXIII-A-G185T polymorphism can explain the hyperfibrinolytic proneness to an early CNV recanalization with a greater visual acuity loss observed in T-allele carriers at the end of follow-up period despite the higher number of PDT-V procedures. In these polymorphic patients, as-needed treated with PDT-Vs in case of persistent activity of neovascular macular degeneration, after each photo-thrombotic procedure the fibrin network of CNV is reliably characterized by an excessive hemodynamic permeation and/or insufficient clot stability to obtain an effective occlusion of the neovascular lesion [50,51,52,55,56] without risk of collateral injuries of the tissues contiguous to the CNV targeted by PDT-V (*i.e*., retina, RPE and choriocapillaris). Retrospective data have documented that the repetitions of verteporfin therapy have induced, in the most part of treated patients, persistent choriocapillary non-perfusion [37,80,81] which, over time, can contribute to the determinism of significant vision loss during standardized photodynamic protocol. This lack of therapeutic responsiveness, despite the high frequency of PDT-V procedures, is consistently related to the absence of an efficient CNV photo-thrombosis, as well as to the presence of iatrogenic damages able to further deteriorate the sub-retinal RPE-Bruch’s membrane barrier of an already degenerated macula.

Further clinico-genetic studies are warranted to verify the possible influence of FXIII-A 185-T-allele in large clusters of patients concomitantly treated with anti-VEGF intravitreal injections and PDT-V for the presence of neovascular macular degenerations. The results of these investigations might be important to outline an appropriate paradigm for guiding and/or personalizing PDT-V in the course of its therapeutic utilization in combination with the widely used anti-VEGF drugs.

## 4. Experimental Section

### 4.1. Study Patients

In the present multicenter study, we conducted a retrospective analysis of the clinical records of Caucasian patients affected by treatment-naive, subfoveal CNV secondary to either AMD or PM. According to the procedures employed during TAP and VIP clinical trials [14,15,16], all selected cases were treated with standardized PDT-V regimen, and were monitored during a 24-month follow-up period. Photodynamic re-treatments were performed when required, in accordance with the international guidelines for PDT-V application, *i.e.*, TAP/VIP protocol: the patients were examined at three-month intervals, and additional courses of treatment were achieved in case of persistence of angiographic dye-leakage from CNV [14,17,47]. The CNV classification was based on the definitions from TAP, VIP, and Visudyne in Minimally Classic Choroidal Neovascularization (VIM) studies [14,15,47,59], including patients with: (1) classic or predominantly classic AMD-related CNV; (2) minimally classic or occult with no classic AMD-related CNV; and (3) classic PM-related CNV. For the purposes of the current investigation, individuals who had correctly and regularly followed a standardized PDT-V protocol were consecutively enrolled. Each included a patient who underwent, within two weeks after the onset of CNV-related visual symptoms, both fluorescein angiography (FA) and indocyanine green angiography (ICGA) to detail the diagnosis of neovascular AMD or neovascular PM. Each photodynamic treatment was performed, within one week after angiographic examinations, according to the guidelines for PDT-V application (TAP/VIP protocol) [14,17,47]. Inclusion and exclusion criteria are summarized in Table 3. All the enrolled patients gave their written informed consent to participate to the study, after a detailed description of the aim-work and of the procedures to be used. Local Ethic Committees reviewed and approved the trial (protocol identification code PRIN-2009NZAZSJ; version of 17 October 2011). The study followed the tenets of the Declaration of Helsinki.

**Table 3:** Inclusion and exclusion criteria.

**Inclusion Criteria**
diagnosis of AMD in Caucasian patients with more than 65 years or of PM in patients with less than 60 years; best-correct visual acuity better than 20/200 (Snellen equivalent); angiographic diagnosis of classic or predominantly classic CNV secondary to AMD, occult with no classic CNV secondary to AMD or classic CNV secondary to PM; active CNV under the geometric center of the foveal avascular zone (subfoveal); greatest linear dimension of the neovascular complex less than 5400 micron in patients with AMD- or PM-related classic CNV; greatest dimension of the neovascular complex equal or less than 4 Macular Photocoagulation Study disc areas in patients with AMD-related minimally classic or occult CNV.
**Exclusion Criteria**
history of any other CNV treatment before and/or during PDT-V protocol; presence of any other possible cause of CNV, such as angioid streaks, chorioretinal inflammatory diseases, hereditary retinal disorders, presumed ocular histoplasmosis syndrome, and/or severe ocular trauma; intraocular surgery and any laser-treatment of the eye during the 6 months before or the 3 months after PDT-V protocol; presence of any significant side effect, condition and/or event possibly influencing the outcome of each PDT-V application; active or chronic systemic diseases related to alterations of hemostatic balance; assumption of any medication known to affect the hemostatic balance, such as antiplatelet, antithrombotic and anticoagulant drugs.

Legend: AMD, age-related macular degeneration; PM, pathologic myopia; CNV, choroidal neovascularization; PDT-V, photodynamic therapy with verteporfin.

### 4.2. Data Collection

In the course of both pre- and post-PDT-V checks, each included patient underwent medical and ophthalmologic histories, auto-refraction, BCVA test, slit-lamp biomicroscopy of the anterior segment, applanation tonometry, 60-diopter lens ophthalmoscopy, FA, and ICGA. Snellen BCVA was measured using a standard logarithmic chart at a test distance of three meters. BCVA values (Snellen equivalent) were converted to the logarithm of the minimum angle of resolution (logMAR) scale for statistical analysis. Soon after the pre-PDT-V angiographies, blood samples were collected for genotyping. Genomic DNA was isolated from peripheral blood by using standard proteinase K treatment followed by phenol-chloroform extraction and ethanol precipitation. In a Peltier Thermal Cycler apparatus (PTC-200, MJ Research, Watertown, MA, USA), samples were polymerase chain reaction (PCR)-genotyped for FXIII-A-G185T variant, employing restriction and electrophoretic analyses [57]. Genotypes for FXIII-A-G185T polymorphism were confirmed by re-genotyping a random selection of samples. All examinations were carried out in a blinded fashion regarding the clinical data of each patient.

### 4.3. Statistical Analyses

The outcome measures were the diversities in: (1) number of PDT-V performed in carriers and non-carriers of FXIII-A 185-T mutated allele during the 24-month therapeutic protocol; and (2) change of BCVA from baseline to 24 months after the first PDT-V among these patients’ categories. Since the typology of the investigated clinical parameters, the sample size calculation, accomplished for the amount of study population (412 cases), provided a value constantly higher than 85%. This test was performed using the PASS 97 statistical program (NCSS Inc., Kaysville, UT, USA).

In the study population, the expected genotype distribution of FXIII-A-G185T polymorphism was checked by the Hardy-Weinberg equilibrium test, and compared with a cluster of normal individuals matched for sex, age and ethnicity with the case group.

Baseline characteristics and outcome measures were compared between FXIII-A 185-T-allele carriers (patients with GT or TT mutated genotypes) and non-carriers (patients with wild-GG genotype). A two-sided *t*-test was employed for comparing the mean of continuous measures, and the *χ^2^* test was used for comparing proportions of categorical measures. A one- or two-way ANOVA procedure was employed to compare, between FXIII-A 185-T-allele carriers and non-carrier, the mean differences in both the total number of photodynamic treatments performed during the 24-month regimen and in the modification of BCVA from baseline to 24 months after the first PDT-V. All analyses were performed by SPSS Statistical Package (SPSS Inc., Chicago, IL, USA). A probability of *p* value <0.05 was considered statistically significant.

## References

[1] Resnikoff S., Pascolini D., Etya’ale D., Kocur I., Pararajasegaram R., Pokharel G.P., Mariotti S.P. (2004). Global data on visual impairment in the year 2002. Bull. World Health Organ..

[2] Klein R., Peto T., Bird A., Vannewkirk M.R. (2004). The epidemiology of age-related macular degeneration. Am. J. Ophthalmol..

[3] Ferris F.L., Fine S.L., Hyman L. (1984). Age-related macular degeneration and blindness due to neovascular maculopathy. Arch. Ophthalmol..

[4] Soubrane G. (2008). Choroidal neovascularization in pathologic myopia: Recent developments in diagnosis and treatment. Surv. Ophthalmol..

[5] Soubrane G., Cruess A., Lotery A., Pauleikhoff D., Monès J., Xu X., Zlateva G., Buggage R., Conlon J., Goss T.F. (2007). Burden and health care resource utilization in neovascular age-related macular degeneration: Findings of a multicountry study. Arch. Ophthalmol..

[6] Colquitt J.L., Jones J., Tan S.C., Takeda A., Clegg A.J., Price A. (2008). Ranibizumab and pegaptanib for the treatment of age-related macular degeneration: A systematic review and economic evaluation. Health Technol. Assess..

[7] Cruess A.F., Zlateva G., Xu X., Soubrane G., Pauleikhoff D., Lotery A., Monès J., Buggage R., Schaefer C., Knight T. (2008). Economic burden of bilateral neovascular age-related macular degeneration: Multi-country observational study. Pharmacoeconomics.

[8] Das A., McGuire P.G. (2003). Retinal and choroidal angiogenesis: Pathophysiology and strategies for inhibition. Prog. Retin. Eye Res..

[9] Bressler S.B. (2009). Introduction: Understanding the role of angiogenesis and antiangiogenic agents in age-related macular degeneration. Ophthalmology.

[10] Chakravarthy U., Soubrane G., Bandello F., Chong V., Creuzot-Garcher C., Dimitrakos S.A., Korobelnik J.F., Larsen M., Monés J., Pauleikhoff D. (2006). Evolving European guidance on the medical management of neovascular age related macular degeneration. Br. J. Ophthalmol..

[11] Wormald R., Evans J., Smeeth L., Henshaw K. (2007). Photodynamic therapy for neovascular age-related macular degeneration. Cochrane Database Syst. Rev..

[12] Vedula S.S., Krzystolik M.G. (2008). Antiangiogenic therapy with anti-vascular endothelial growth factor modalities for neovascular age-related macular degeneration. Cochrane Database Syst. Rev..

[13] Cohen S.Y. (2009). Anti-VEGF drugs as the 2009 first-line therapy for choroidal neovascularization in pathologic myopia. Retina.

[14] Treatment of Age-Related Macular Degeneration with Photodynamic Therapy (TAP) Study Group (1999). Photodynamic therapy of subfoveal choroidal neovascularization in age-related macular degeneration with verteporfin: One-year results of 2 randomized clinical trials—TAP Report No. 1. Arch. Ophthalmol..

[15] Verteporfin in Photodynamic Therapy (VIP) Study Group (2001). Verteporfin therapy of subfoveal choroidal neovascularization in age related macular degeneration: 2 year results of a randomized clinical trial including lesions with occult with no classic choroidal neovascularization—VIP Report No. 2. Am. J. Ophthalmol..

[16] Blinder K.J., Blumenkranz M.S., Bressler N.M., Bressler S.B., Donato G., Lewis H., Lim J.I., Menchini U., Miller J.W., Monès J.M., Verteporfin in Photodynamic Therapy (VIP) Study Group (2003). Verteporfin therapy of subfoveal choroidal neovascularization in pathologic myopia: 2-Year results of a randomized clinical trial—VIP Report No. 3. Ophthalmology.

[17] Verteporfin Roundtable Participants (2005). Guidelines for using verteporfin (Visudyne) in photodynamic therapy for choroidal neovascularization due to age-related macular degeneration and other causes: Update. Retina.

[18] Rosenfeld P.J., Brown D.M., Heier J.S., Boyer D.S., Kaiser P.K., Chung C.Y., Kim R.Y., MARINA Study Group (2006). Ranibizumab for neovascular age-related macular degeneration. N. Engl. J. Med..

[19] Mitchell P., Korobelnik J.F., Lanzetta P., Holz F.G., Prünte C., Schmidt-Erfurth U., Tano Y., Wolf S. (2010). Ranibizumab (Lucentis) in neovascular age-related macular degeneration: Evidence from clinical trials. Br. J. Ophthalmol..

[20] Martin D.F., Maguire M.G., Fine S.L., Ying G.S., Jaffe G.J., Grunwald J.E., Toth C., Redford M., Ferris F.L., Comparison of Age-related Macular Degeneration Treatments Trials (CATT) Research Group (2012). Ranibizumab and bevacizumab for treatment of neovascular age-related macular degeneration: Two-year results. Ophthalmology.

[21] Heier J.S., Brown D.M., Chong V., Korobelnik J.F., Kaiser P.K., Nguyen Q.D., Kirchhof B., Ho A., Ogura Y., Yancopoulos G.D., VIEW 1 and VIEW 2 Study Groups (2012). Intravitreal aflibercept (VEGF trap-eye) in wet age-related macular degeneration. Ophthalmology.

[22] Chakravarthy U., Harding S.P., Rogers C.A., Downes S.M., Lotery A.J., Culliford L.A., Reeves B.C., IVAN Study Investigators (2013). Alternative treatments to inhibit VEGF in age-related choroidal neovascularisation: 2-year findings of the IVAN randomised controlled trial. Lancet.

[23] Lazic R., Gabric N. (2007). Verteporfin therapy and intravitreal bevacizumab combined and alone in choroidal neovascularization due to age-related macular degeneration. Ophthalmology.

[24] Antoszyk A.N., Tuomi L., Chung C.Y., Singh A., FOCUS Study Group (2008). Ranibizumab combined with verteporfin photodynamic therapy in neovascular age-related macular degeneration (FOCUS): Year 2 results. Am. J. Ophthalmol..

[25] Navea A., Mataix J., Desco M.C., Garcia-Pous M., Palacios E. (2009). One-year follow-up of combined customized therapy. Photodynamic therapy and bevacizumab for exudative age-related macular degeneration. Retina.

[26] Kaiser P.K., Boyer D.S., Garcia R., Hao Y., Hughes M.S., Jabbour N.M., Kaiser P.K., Mieler W., Slakter J.S., Samuel M. (2009). Verteporfin photodynamic therapy combined with intravitreal bevacizumab for neovascular age-related macular degeneration. Ophthalmology.

[27] Desco M.C., Mataix J., Garcia-Pous M., Palacios-Pozo E., Navea A. (2011). Combined therapy: Photodynamic therapy and bevacizumab to treat myopic neovascular membranes. One-year follow-up. Retina.

[28] Bashshur Z.F., Schakal A.R., El-Mollayess G.M., Arafat S., Jaafar D., Salti H.I. (2011). Ranibizumab monotherapy *versus* single-session verteporfin photodynamic therapy combined with as-needed ranibizumab treatment for the management of neovascular age-related macular degeneration. Retina.

[29] Kaiser P.K., Boyer D.S., Cruess A.F., Slakter J.S., Pilz S., Weisberger A., DENALI Study Group (2012). Verteporfin plus ranibizumab for choroidal neovascularization in age-related macular degeneration: twelve-month results of the DENALI study. Ophthalmology.

[30] Krebs I., Vécsei Marlovits V., Bodenstorfer J., Glittenberg C., Ansari Shahrezaei S., Ristl R., Binder S. (2013). Comparison of Ranibizumab monotherapy *versus* combination of Ranibizumab with photodynamic therapy with neovascular age-related macular degeneration. Acta Ophthalmol..

[31] Schmidt-Erfurth U.M., Prünte C. (2007). Management of neovascular age-related macular degeneration. Prog. Retin. Eye Res..

[32] Shah G.K., Sang D.N., Hughes M.S. (2009). Verteporfin combination regimens in the treatment of neovascular age-related macular degeneration. Retina.

[33] Schmidt-Erfurth U., Kiss C., Sacu S. (2009). The role of choroidal hypoperfusion associated with photodynamic therapy in neovascular age-related macular degeneration and the consequences for combination strategies. Prog. Retin. Eye Res..

[34] Amoaku W.M., Chakravarthy U., Gale R., Gavin M., Ghanchi F., Gibson J., Harding S., Johnston R.L., Kelly S., Lotery A. (2015). Defining response to anti-VEGF therapies in neovascular AMD. Eye.

[35] Schmidt-Erfurth U., Hasan T. (2000). Mechanisms of action of photodynamic therapy with verteporfin for the treatment of age-related macular degeneration. Surv. Ophthalmol..

[36] Schlotzer-Schrehardt U., Viestenz A., Naumann G.O., Laqua H., Michels S., Schmidt-Erfurth U. (2002). Dose-related structural effects of photodynamic therapy on choroidal and retinal structures of human eyes. Graefes Arch. Clin. Exp. Ophthalmol..

[37] Schmidt-Erfurth U., Michels S., Barbazetto I., Laqua H. (2002). Photodynamic effects on choroidal neovascularization and physiological choroid. Investig. Ophthalmol. Vis. Sci..

[38] Michels S., Schmidt-Erfurth U. (2003). Sequence of early vascular events after photodynamic therapy. Investig. Ophthalmol. Vis. Sci..

[39] Schmidt-Erfurth U., Niemeyer M., Geitzenauer W., Michels S. (2005). Time course and morphology of vascular effects associated with photodynamic therapy. Ophthalmology.

[40] Blinder K.J., Bradley S., Bressler N.M., Bressler S.B., Donati G., Hao Y., Ma C., Menchini U., Miller J., Potter M.J. (2003). Effect of lesion size, visual acuity, and lesion composition on visual acuity change with and without verteporfin therapy for choroidal neovascularization secondary to age-related macular degeneration: TAP and VIP report 1. Am. J. Ophthalmol..

[41] Axer-Siegel R., Ehrlich R., Yassur Y., Rosenblatt I., Kramer M., Priel E., Benjamini Y., Weinberger D. (2004). Photodynamic therapy for age-related macular degeneration in a clinical setting: Visual results and angiographic patterns. Am. J. Ophthalmol..

[42] Arias L., Pujol O., Berniell J., Rubio M., Roca G., Castillo L., Acebes E. (2005). Impact of lesion size on photodynamic therapy with verteporfin of predominantly classic lesions in age related macular degeneration. Br. J. Ophthalmol..

[43] Ergun E., Heinzl H., Stur M. (2004). Prognostic factors influencing visual outcome of photodynamic therapy for subfoveal choroidal neovascularization in pathologic myopia. Am. J. Ophthalmol..

[44] Axer-Siegel R., Ehrlich R., Weinberger D., Rosenblatt I., Shani L., Yassur Y., Priel E., Kramer M. (2004). Photodynamic therapy of subfoveal choroidal neovascularization in high myopia in a clinical setting: Visual outcome in relation to age at treatment. Am. J. Ophthalmol..

[45] Chan W.M., Lai T.Y., Tano Y., Liu D.T., Li K.K., Lam D.S. (2006). Photodynamic therapy in macular diseases of Asian populations: When East meets West. Jpn. J. Ophthalmol..

[46] Yang N., Fan C.M., Ho C.K. (2006). Review of first year result of photodynamic therapy on age-related macular degeneration in Chinese population. Eye.

[47] Verteporfin in Photodynamic Therapy (VIP) Study Group (2001). Photodynamic therapy of subfoveal choroidal neovascularization in pathologic myopia with verteporfin: 1-year results of a randomized clinical trial—VIP report No. 1. Ophthalmology.

[48] Lam D.S., Chan W.M., Liu D.T., Fan D.S., Lai W.W., Chong K.K. (2004). Photodynamic therapy with verteporfin for subfoveal choroidal neovascularisation of pathologic myopia in Chinese eyes: A prospective series of 1 and 2 year follow up. Br. J. Ophthalmol..

[49] Hayashi K., Ohno-Matsui K., Teramukai S., Shimada N., Moriyama M., Hara W., Yoshida T., Tokoro T., Mochizuki M. (2008). Photodynamic therapy with verteporfin for choroidal neovascularization of pathologic myopia in Japanese patients: Comparison with nontreated controls. Am. J. Ophthalmol..

[50] Parmeggiani F., Costagliola C., Gemmati D., D’Angelo S., Perri P., Scapoli G.L., Catozzi L., Federici F., Sebastiani A., Incorvaia C. (2007). Predictive role of coagulation-balance gene polymorphisms in the efficacy of photodynamic therapy with verteporfin for classic choroidal neovascularization secondary to age-related macular degeneration. Pharmacogenet. Genom..

[51] Parmeggiani F., Costagliola C., Gemmati D., D’Angelo S., Perri P., Campa C., Catozzi L., Federici F., Sebastiani A., Incorvaia C. (2008). Coagulation-balance genetic predictors for efficacy of photodynamic therapy in occult choroidal neovascularization secondary to age-related macular degeneration. Investig. Ophthalmol. Vis. Sci..

[52] Parmeggiani F., Gemmati D., Costagliola C., Semeraro F., D’Angelo S., Perri P., Sebastiani A., Incorvaia C. (2010). Impact of coagulation-balance gene predictors on efficacy of photodynamic therapy for choroidal neovascularization in pathologic myopia. Ophthalmology.

[53] Parmeggiani F., Gemmati D., Costagliola C., Sebastiani A., Incorvaia C. (2009). Predictive role of gene polymorphisms affecting thrombin-generation pathway in variable efficacy of photodynamic therapy for neovascular age-related macular degeneration. Recent. Pat. DNA Gene Seq..

[54] Parmeggiani F., Gemmati D., Costagliola C., Sebastiani A., Incorvaia C. (2009). Predictive role of C677T MTHFR polymorphism in variable efficacy of photodynamic therapy for neovascular age-related macular degeneration. Pharmacogenomics.

[55] Parmeggiani F., Costagliola C., Incorvaia C., Sebastiani A., Gemmati D. (2011). Pharmacogenetic aspects in therapeutic management of subfoveal choroidal neovascularisation: Role of factor XIII-A 185 T-allele. Curr. Drug Targets.

[56] Parmeggiani F., Gemmati D., Costagliola C., Semeraro F., Perri P., D’Angelo S., Romano M.R., de Nadai K., Sebastiani A., Incorvaia C. (2011). Genetic predictors of response to photodynamic therapy. Mol. Diagn. Ther..

[57] Gemmati D., Serino M.L., Ongaro A., Tognazzo S., Moratelli S., Resca R., Moretti M., Scapoli G.L. (2001). A common mutation in the gene for coagulation factor XIII-A (VAL34Leu): A risk factor for primary intracerebral hemorrhage is protective against atherothrombotic diseases. Am. J. Hematol..

[58] Bressler N.M., TAP Study Group (2001). Photodynamic therapy of subfoveal choroidal neovascularization in age-related macular degeneration with verteporfin: Two-year results of 2 randomized clinical trials—TAP report No. 2. Arch. Ophthalmol..

[59] Azab M., Boyer D.S., Bressler N.M., Bressler S.B., Cihelkova I., Hao Y., Immonen I., Lim J.I., Menchini U., Naor J. (2005). Verteporfin therapy of subfoveal minimally classic choroidal neovascularization in age-related macular degeneration: 2-Year results of a randomized clinical trial. Arch. Ophthalmol..

[60] Campa C., Costagliola C., Incorvaia C., Sheridan C., Semeraro F., De Nadai K., Sebastiani A., Parmeggiani F. (2010). Inflammatory mediators and angiogenic factors in choroidal neovascularization: pathogenetic interactions and therapeutic implications. Mediators Inflamm..

[61] Parmeggiani F., Romano M.R., Costagliola C., Semeraro F., Incorvaia C., D’Angelo S., Perri P., de Palma P., de Nadai K., Sebastiani A. (2012). Mechanism of inflammation in age-related macular degeneration. Mediators Inflamm..

[62] Parmeggiani F., Sorrentino F.S., Romano M.R., Costagliola C., Semeraro F., Incorvaia C., D’Angelo S., Perri P., de Nadai K., Bonomo Roversi E. (2013). Mechanism of inflammation in age-related macular degeneration: An up-to-date on genetic landmarks. Mediators Inflamm..

[63] Gallenga C.E., Parmeggiani F., Costagliola C., Sebastiani A., Gallenga P.E. (2014). Inflammaging: Should this term be suitable for age related macular degeneration too?. Inflamm. Res..

[64] Incorvaia C., Campa C., Parmeggiani F., Menzione M., D’Angelo S., Della Corte M., Rinaldi M., Romano M., Dell’Omo R., Costagliola C. (2008). 12-month retrospective study and review of photodynamic therapy with verteporfin for subfoveal choroidal neovascularization in age-related macular degeneration. Retina.

[65] Costagliola C., Campa C., Incorvaia C., Parmeggiani F., Menzione M., Della Corte M., Rinaldi M., Romano M., Semeraro F. (2008). Verteporfin photodynamic therapy for subfoveal choroidal neovascularization in pathologic myopia: A 12-month retrospective review. Eur. J. Ophthalmol..

[66] Ichinose A. (2001). Physiopathology and regulation of factor XIII. Thromb. Haemost..

[67] Bereczky Z., Katona E., Muszbek L. (2003). Fibrin stabilization (factor XIII), fibrin structure and thrombosis. Pathophysiol. Haemost. Thromb..

[68] Mosesson M.W. (2005). Fibrinogen and fibrin structure and functions. J. Thromb. Haemost..

[69] Jámbor C., Reul V., Schnider T.W., Degiacomi P., Metzner H., Korte W.C. (2009). *In vitro* inhibition of factor XIII retards clot formation, reduces clot firmness, and increases fibrinolytic effects in whole blood. Anesth. Analg..

[70] Anwar R., Gallivan L., Edmonds S.D., Markham A.F. (1999). Genotype/phenotype correlations for coagulation factor XIII: Specific normal polymorphisms are associated with high or low factor XIII specific activity. Blood.

[71] De Lange M., Andrew T., Snieder H., Ge D., Futers T.S., Standeven K., Spector T.D., Grant P.J., Ariëns R.A. (2006). Joint linkage and association of six single-nucleotide polymorphisms in the factor XIII-A subunit gene point to V34L as the main functional locus. Arterioscler. Thromb. Vasc. Biol..

[72] Kohler H.P., Ariëns R.A., Whitaker P., Grant P.J. (1998). A common coding polymorphism in the FXIII A-subunit gene (*FXIIIVal34Leu*) affects cross-linking activity. Thromb. Haemost..

[73] Ariëns R.A., Philippou H., Nagaswami C., Weisel J.W., Lane D.A., Grant P.J. (2000). The factor XIII V34L polymorphism accelerates thrombin activation of factor XIII and affects cross-linked fibrin structure. Blood.

[74] Maurer M.C., Trumbo T.A., Isetti G., Turner B.T. (2006). Probing interactions between the coagulants thrombin, Factor XIII, and fibrin(ogen). Arch. Biochem. Biophys..

[75] Schroeder V., Kohler H.P. (2000). Effect of factor XIII Val34Leu on α2-antiplasmin incorporation into fibrin. Thromb. Haemost..

[76] Ariëns R.A., Lai T.S., Weisel J.W., Greenberg C.S., Grant P.J. (2002). Role of factor XIII in fibrin clot formation and effects of genetic polymorphisms. Blood.

[77] Lim B.C., Ariëns R.A., Carter A.M., Weisel J.W., Grant P.J. (2003). Genetic regulation of fibrin structure and function: Complex gene-environment interactions may modulate vascular risk. Lancet.

[78] Kobbervig C., Williams E. (2004). FXIII polymorphisms, fibrin clot structure and thrombotic risk. Biophys. Chem..

[79] Komáromi I., Bagoly Z., Muszbek L. (2011). Factor XIII: Novel structural and functional aspects. J. Thromb. Haemost..

[80] Durlu Y.K. (2003). Retinal and choroidal alterations following photodynamic therapy. Adv. Exp. Med. Biol..

[81] Dewi N.A., Yuzawa M., Tochigi K., Kawamura A., Mori R. (2008). Effects of photodynamic therapy on the choriocapillaris and retinal pigment epithelium in the irradiated area. Jpn. J. Ophthalmol..

